# Which Species Are We Researching and Why? A Case Study of the Ecology of British Breeding Birds

**DOI:** 10.1371/journal.pone.0131004

**Published:** 2015-07-08

**Authors:** Ailsa J. McKenzie, Peter A. Robertson

**Affiliations:** 1 Centre for Wildlife Management, School of Biology, Newcastle University, Ridley Building, Claremont Road, Newcastle upon Tyne, United Kingdom; 2 National Wildlife Management Centre, Animal and Plant Health Agency, Sand Hutton, York, United Kingdom; University of Lleida, SPAIN

## Abstract

Our ecological knowledge base is extensive, but the motivations for research are many and varied, leading to unequal species representation and coverage. As this evidence is used to support a wide range of conservation, management and policy actions, it is important that gaps and biases are identified and understood. In this paper we detail a method for quantifying research effort and impact at the individual species level, and go on to investigate the factors that best explain between-species differences in outputs. We do this using British breeding birds as a case study, producing a ranked list of species based on two scientific publication metrics: total number of papers (a measure of research quantity) and h-index (a measure of the number of highly cited papers on a topic – an indication of research quality). Widespread, populous species which are native, resident and in receipt of biodiversity action plans produced significantly higher publication metrics. Guild was also significant, birds of prey the most studied group, with pigeons and doves the least studied. The model outputs for both metrics were very similar, suggesting that, at least in this example, research quantity and quality were highly correlated. The results highlight three key gaps in the evidence base, with fewer citations and publications relating to migrant breeders, introduced species and species which have experienced contractions in distribution. We suggest that the use of publication metrics in this way provides a novel approach to understanding the scale and drivers of both research quantity and impact at a species level and could be widely applied, both taxonomically and geographically.

## Introduction

The knowledge base for wildlife ecology is extensive, however with research motivations many and varied, the representation of species within this knowledge base is unequal, not only in terms of species and subject area, but also with respect to paper “quality” or “impact” (e.g. citation record) [1–6]. With many ecological studies used to guide species conservation, management and policy it is important that knowledge gaps are identified and understood. In this paper we describe a novel method for the quantification of research effort and impact at the individual species level, and also present an investigation of the factors that best explain between-species differences in outputs. While a body of literature on research effort between and within species does exist [1–6] this is one of the first papers to also include an estimate of scientific “impact”. We illustrate this method using British breeding birds as a case study. British breeding birds provide an excellent test case as they are a very well-studied group, with a varied range of research motivations including ease of study (many species are common and easy to study both in the wild and captivity), a largely positive public perception (the Royal Society for the Protection of Birds is the largest membership environment organisation in Europe [7]), interests of individual researchers (many researchers, or research groups have studied individual species over many decades e.g. Great Tits *Parus major* [8]), and changes in conservation status.

## Materials and Methods

Two pre-existing publication metrics were selected for use–total number of papers per species (a measure of research volume) and species h-index (an indication of volume PLUS “quality”). Developed by Hirsch [9] as a means of measuring the impact and sustainability of scientific output of individual researchers [10], h-index differs from other publication metrics in that it highlights papers which are regarded by fellow scientists as worthy of citation. This paper uses the h-index approach in a novel way—to assess the volume and impact of papers about British breeding bird species, using “individual species” in place of “individual researcher”.

225 species were included in the analysis–all species classed as breeding by the British Ornithologists Union (resident breeder, introduced breeder, migrant breeder, has bred, may have bred [11], plus two additional species (Eagle owl *Bubo bubo* and Monk parakeet *Myiopsitta monachus*). While not officially classed as breeding, these species are widely regarded as doing so, and are known to have conflicts with human interests [12]. A search was made for the scientific name of each species using Thomson Reuters Web of Science. Scientific names were used for these searches as many common bird names carry alternative meanings in English which complicate their use. Results were then refined by predefined Web of Science Research Domain (“Science Technology”), Research Areas (“Zoology”, “Environmental Sciences Ecology” and “Biodiversity Conservation”) and Countries/Territories (“UK”, “England”, “Scotland” and “Wales”). The decision was taken not to include Ireland (Northern or Southern) as preliminary work found there to be insufficient discrimination between the areas for them to be included accurately. The remaining records, including all publication information (e.g. title, abstract, authors, source, publication date), were then imported into Excel.

Each paper was checked for relevance using the criteria in Table 1. In the majority of cases (around 80%), information already contained in the database (title, abstract, source) was sufficient to make these judgements. However, when this information was inadequate the full paper was sourced. It is possible that by following the criteria laid out in Box 1, some relevant papers were excluded from the analysis (i.e. papers which did not specify the scientific name of the target species in the abstract or title). To determine the extent of this issue we carried out a subsetting exercise, where 10% of previously excluded papers (those which were extracted from WoS but did not meet the Box 1 criteria) were rechecked for relevance. It was found that only between 0.7 and 1.4% of relevant papers had been excluded as a result of the Box 1 criteria.

**Table 1:** Paper qualifying criteria.

To qualify, a paper must:
1) Feature the target species in the title or abstract
2) Be carried out, at least in part, in Great Britain (studies carried out solely in Northern or Southern Ireland were excluded)
3) Be primarily ecological, dealing directly with or referring specifically to free-living bird populations. Papers dealing solely with anatomy, genetics or captive/laboratory animal studies were excluded, unless authors directly related observations to the ecology of the populations of interest.

The default timespan was used for all searches (1864 to 2014, however all papers which met the relevancy criteria were published between 1972 and 2014. The remaining papers were then sorted by decreasing number of citations (“Times Cited”) and h-index calculated. The h-index was calculated as the largest number *h* such that *h* publications have at least *h* citations [9]. For example, if a species had three associated publications, cited 10, seven and two times, it would have an h-index of two, as two papers attracted two or more citations. The total number of papers per species was also calculated based on the total number of papers identified by the search.

A range of covariates deemed likely to influence species publication metrics were collated from a variety of sources and entered into the database along with the publication metrics (see S1 Table, for full list and sources). Simple statistical comparisons (Kruskal Wallis tests as data were not normally distributed) were initially made between publication metrics and a range of key covariates. Native/introduced status [13], Biodiversity Action Plan (BAP; [14]) and IUCN Red List status [15] and breeding status [11] were included as two level factors (native vs. introduced, BAP vs. non-BAP, Red List status “Not assessed” or “Least concern” vs. “Near threatened”, resident or migrant). 20 and 40 year distribution trends were included as three level factors (increase, stable, decline; [16]) while guild was included as an 11 level factor—ducks and geese (22 species), herons/bitterns/egrets (10 species), gamebirds (10 species), corvids and small passerines (6 and 80 spp), birds of prey (21 species), seabirds (23 species), doves and pigeons (6 species), grebes/divers/rails (14 species) and waders (24 species) and other (9 species) [13].

Publication metrics for each species were subsequently entered as the response variable into general linear models (GLMs) in R (version 3.0.0) along with a suite of predictors based on a combination of *a priori* predictions and data exploration. To this end, population size [13](or distribution [16]) was included in the model (these were correlated—Pearson’s coefficient = 0.508, p<0.001—although population size provided a slightly better fit to the data than distribution; both log10+1 transformed) along with factors describing whether a species was native or introduced, the subject of a BAP, its Red List status, its breeding status, its 20 year distribution trend and guild. Weight was also a good predictor in exploratory models [16,17]; however this was confounded by guild, which provided a better fit for the data.

## Results

**Fig 1:**
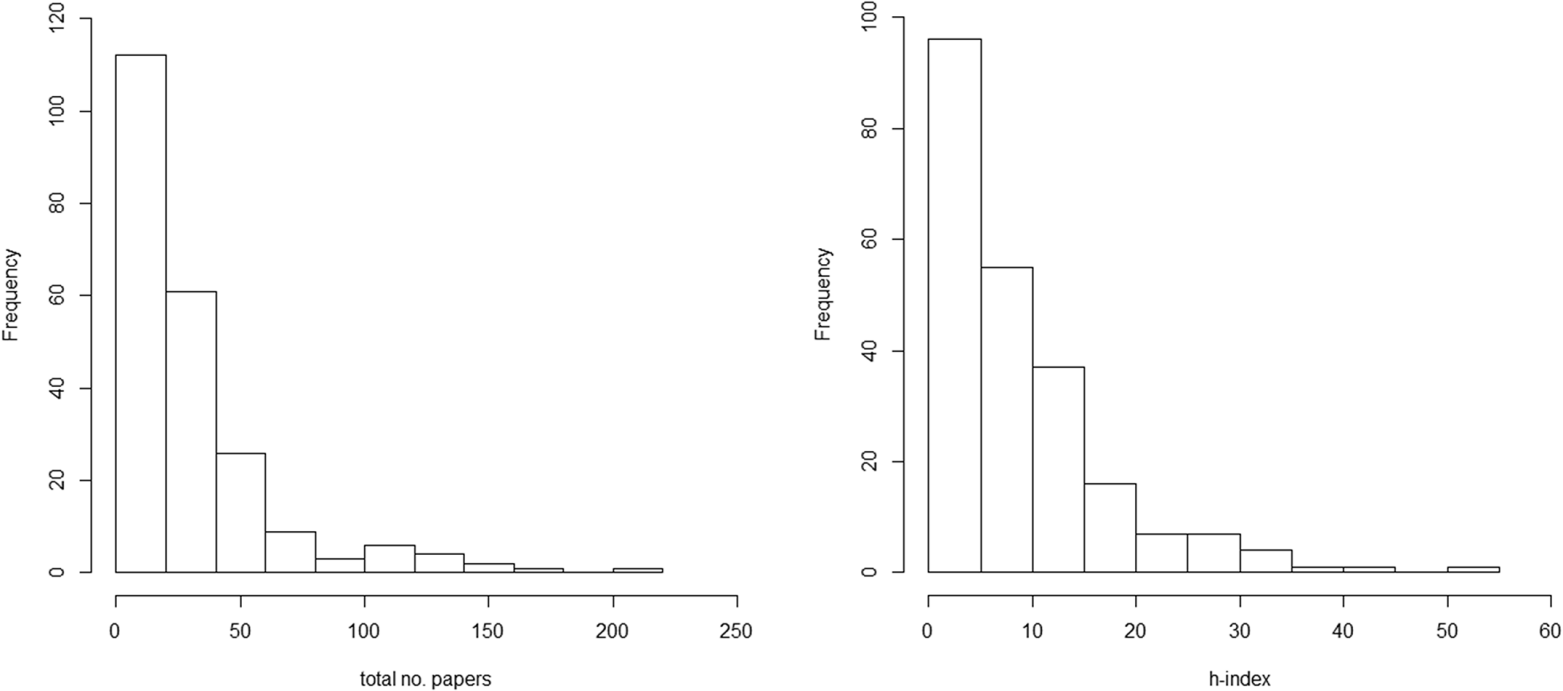
Histograms of the distribution of publication metrics for individual species a) total number of papers; b) h-index.

Of the 11,559 papers extracted from Web of Science, 6716 relevant science references were identified based on 5816 publications (some references referred to more than one species; mean 2.8 species). The publication years of these papers ranged from 1972 to 2014. Together these 5016 references attracted 28,091 citations and had an overall h-index of 111. The full list of species and their publication metrics is shown in Table 2. Total number of papers for the 225 species ranged from 0 (n = 4) to 213 (n = 1) and h-indices from 0 (n = 20) to 52 (n = 1) (Fig 1).

**Table 2:** Publication metrics for British breeding birds: h-index (a measure of quality) and total number of papers (a measure of quantity).

Scientific name	Common name	Total no. papers	h-index
*Parus major*	Great Tit	209	52
*Lagopus lagopus*	Red (Willow) Grouse	165	33
*Haematopus ostralegus*	Eurasian Oystercatcher	156	33
*Accipiter nisus*	Eurasian Sparrowhawk	141	30
*Sturnus vulgaris*	Starling	137	41
*Cyanistes caeruleus (Parus caeruleus)*	Blue Tit	129	36
*Uria aalge*	Guillemot	128	33
*Vanellus vanellus*	Lapwing	121	26
*Alauda arvensis*	Skylark	118	31
*Tringa tetanus*	Redshank	114	30
*Turdus merula*	Blackbird	106	29
*Phalacrocorax carbo*	Cormorant	105	14
*Circus cyaneus*	(Northern) Hen Harrier	104	20
*Passer domesticus*	House Sparrow	102	26
*Rissa tridactyla*	Kittiwake	93	26
*Falco peregrinus*	Peregrine Falcon	84	15
*Larus argentatus*	Herring Gull	81	20
*Cygnus olor*	Mute Swan	75	13
*Stercorarius skua (Catharacta skua)*	Great Skua	70	23
*Emberiza citrinella*	Yellowhammer	69	25
*Perdix perdix*	Grey Partridge	68	20
*Hirundo rustica*	Barn Swallow	66	17
*Fringilla coelebs*	Chaffinch	64	20
*Phalacrocorax aristotelis*	Shag	62	26
*Pluvialis apricaria (Pluvialis apricarius)*	Golden Plover	62	18
*Buteo buteo*	Buzzard	61	17
*Larus fuscus*	Lesser Black-backed Gull	58	19
*Morus bassanus (Sula bassanus)*	Northern Gannet	58	13
*Aquila chrysaetos*	Golden Eagle	57	15
*Falco columbarius*	Merlin	56	10
*Puffinus puffinus*	Manx Shearwater	56	15
*Corvus frugilegus*	Rook	55	14
*Phasianus colchicus*	Pheasant	55	15
*Tyto alba*	Barn Owl	54	13
*Prunella modularis*	Dunnock	53	25
*Tetrao tetrix (Lyrurus tetrix)*	Black Grouse	52	14
*Acrocephalus scirpaceus*	Eurasian Reed Warbler	51	22
*Emberiza calandra (Miliaria calandra)*	Corn Bunting	51	19
*Erithacus rubecula*	European Robin	51	21
*Fratercula arctica*	Puffin	51	19
*Calidris alpine (Erolia alpine)*	Dunlin	50	16
*Strix aluco*	Tawny Owl	50	19
*Corvus corone*	Carrion Crow	49	14
*Cuculus canorus*	Common Cuckoo	49	24
*Numenius arquata*	Eurasian Curlew	48	13
*Aegithalos caudatus*	Long-tailed Tit	46	21
*Ardea cinerea*	Grey Heron	46	10
*Fulmarus glacialis*	Northern Fulmar	46	16
*Falco tinnunculus*	Eurasian Kestrel	45	15
*Tetrao urogallus*	Capercaillie	45	15
*Pica pica*	Magpie	42	19
*Cinclus cinclus*	Dipper	41	19
*Columba palumbus*	Woodpigeon	40	10
*Pyrrhocorax pyrrhocorax*	Chough	40	11
*Corvus corax*	Common Raven	38	7
*Hydrobates pelagicus (Thalassidroma pelagicus)*	European Storm Petrel	38	6
*Milvus milvus*	Red Kite	38	7
*Anser anser*	Greylag Goose	37	6
*Turdus philomelos*	Song Thrush	37	16
*Delichon urbicum Delichon urbica*	Common House Martin	36	14
*Chroicocephalus ridibundus (Larus ridibundus)*	Black-headed Gull	35	8
*Crex crex*	Corncrake	33	13
*Mergus merganser*	Goosander	33	5
*Dendrocopos major (Dryobates major)*	Great Spotted Woodpecker	32	8
*Ficedula hypoleuca*	European Pied Flycatcher	32	12
*Gallinula chloropus*	Common Moorhen	32	13
*Troglodytes troglodytes*	Wren	32	15
*Alca torda*	Razorbill	31	14
*Anthus pratensis*	Meadow Pipit	31	11
*Apus apus (Micropus apus)*	Common Swift	31	7
*Caprimulgus europaeus*	European Nightjar	31	7
*Garrulus glandarius*	Jay	31	11
*Poecile palustris (Parus palustris)*	Marsh Tit	31	13
*Somateria mollissima*	Eider	31	10
*Arenaria interpres*	Ruddy Turnstone	30	12
*Gallinago gallinago (Capella gallinago)*	Common Snipe	30	12
*Actitis hypoleucos (Tringa hypoleucos)*	Common Sandpiper	28	10
*Anas platyrhynchos*	Mallard	28	11
*Charadrius hiaticula*	Common Ringed Plover	28	11
*Oxyura jamaicensis*	Ruddy Duck	28	2
*Riparia riparia*	Sand Martin	28	9
*Stercorarius parasiticus*	Arctic Skua	28	12
*Botaurus stellaris*	Eurasian Bittern	27	8
*Emberiza schoeniclus*	Common Reed Bunting	27	12
*Falco subbuteo*	Eurasian Hobby	27	4
*Larus canus*	Common Gull	27	5
*Muscicapa striata*	Spotted Flycatcher	27	9
*Sterna paradisaea*	Arctic Tern	27	10
*Haliaeetus albicilla*	White-tailed Eagle	26	5
*Larus marinus*	Great Black-backed Gull	26	5
*Passer montanus*	Tree Sparrow	26	10
*Sterna hirundo*	Common Tern	26	10
*Periparus ater (Parus ater)*	Coal Tit	25	10
*Acrocephalus schoenobaenus*	Sedge Warbler	24	13
*Carduelis chloris (Chloris chloris)*	European Greenfinch	24	10
*Mergus serrator*	Red-breasted Merganser	24	4
*Loxia curvirostra*	Common (red) Crossbill	23	8
*Phylloscopus trochilus*	Willow Warbler	23	10
*Branta canadensis*	Canada Goose	22	5
*Carduelis cannabina (Acanthis cannabina)*	Linnet	22	13
*Corvus monedula*	Jackdaw	22	10
*Lullula arborea*	Woodlark	22	7
*Luscinia megarhynchos*	Common Nightingale	22	10
*Sylvia atricapilla*	Blackcap	22	9
*Sylvia undata (Melizophilus undatas)*	Dartford Warbler	22	8
*Turdus torquatus*	Ring Ouzel	22	6
*Anas penelope (Mareca penelope)*	Eurasian Wigeon	21	7
*Motacilla flava*	Western Yellow Wagtail	21	10
*Oenanthe oenanthe*	Wheatear	21	12
*Phylloscopus collybita*	Chiffchaff	21	10
*Pyrrhula pyrrhula*	Bullfinch	21	12
*Streptopelia turtur*	Turtle Dove	21	10
*Tadorna tadorna*	Shelduck	21	11
*Carduelis flavirostris (Acanthis flavirostris)*	Twite	20	4
*Cygnus cygnus*	Whooper Swan	20	6
*Fulica atra*	Eurasian Coot	20	4
*Gavia stellata (Colymbus stellata)*	Red-throated Diver	20	4
*Saxicola rubicola (Saxicola torquata)*	Stonechat	20	9
*Scolopax rusticola*	Woodcock	20	6
*Aythya fuligula (Nyroca fuligula)*	Tufted Duck	19	10
*Emberiza cirlus*	Cirl Bunting	19	9
*Asio flammeus*	Short-eared Owl	18	6
*Charadrius morinellus (Eudromias morinellus)*	Dotterel	18	9
*Columba livia*	Rock Dove	18	6
*Limosa limosa*	Black-tailed Godwit	18	11
*Motacilla alba*	Pied Wagtail	18	7
*Sylvia communis*	Whitethroat	18	11
*Podiceps auritus*	Slavonian Grebe	17	3
*Alectoris rufa*	Red-legged Partridge	16	8
*Burhinus oedicnemus (Oedicnemus oedicnemus)*	Stone-curlew	16	10
*Lagopus muta (Lagopus mutus)*	Ptarmigan	16	6
*Melanitta nigra*	Common (Black) Scoter	16	2
*Oceanodroma leucorhoa*	Leach's Petrel	16	3
*Pernis apivorus*	Honey-buzzard	16	1
*Sitta europaea*	Nuthatch	16	6
*Accipiter gentilis (Astur gentilis)*	Northern Goshawk	15	7
*Aythya ferina (Nyroca farina)*	Pochard	15	6
*Charadrius dubius*	Little Ringed Plover	15	1
*Certhia familiaris*	Treecreeper	14	4
*Egretta garzetta*	Little Egret	14	2
*Gavia arctica (Colymbus arctica)*	Black-throated Diver	14	5
*Loxia scotica*	Scottish Crossbill	14	6
*Numenius phaeopus*	Whimbrel	13	6
*Sylvia curruca*	Lesser Whitethroat	13	2
*Cepphus grylle*	Black Guillemot	12	6
*Pandion haliaetus*	Osprey	12	3
*Picus viridis*	Green Woodpecker	12	4
*Plectrophenax nivalis*	Snow Bunting	12	6
*Sterna sandvicensis*	Sandwich Tern	12	4
*Streptopelia decaocto*	Collared Dove	12	5
*Bucephala clangula (Glaucionetta clangula)*	Common Goldeneye	11	3
*Carduelis spinus (Spinus spinus)*	Eurasian Siskin	11	4
*Motacilla cinerea*	Grey Wagtail	11	6
*Tachybaptus ruficollis (Podiceps ruficollis)*	Little Grebe	11	2
*Turdus viscivorus*	Mistle Thrush	11	5
*Alcedo atthis*	Common Kingfisher	10	2
*Asio otus*	Long-eared Owl	10	3
*Circus aeruginosus*	Western Marsh Harrier	10	2
*Coccothraustes coccothraustes*	Hawfinch	10	1
*Dendrocopos minor (Dryobates minor)*	Lesser Spotted Woodpecker	10	3
*Phoenicurus phoenicurus*	Redstart	10	6
*Recurvirostra avosetta*	Avocet	10	2
*Psittacula krameri*	Ring-necked Parakeet	9	4
*Regulus regulus*	Goldcrest	9	3
*Saxicola rubetra*	Whinchat	9	3
*Tringa nebularia*	Greenshank	9	3
*Anas crecca (Querquedula crecca)*	Common Teal	8	4
*Anthus trivialis*	Tree Pipit	8	4
*Athene noctua (Carine noctua)*	Little Owl	8	2
*Cettia cetti*	Cetti's Warbler	8	3
*Locustella naevia*	Eastern Grasshopper Warbler	8	3
*Lophophanes cristatus (Parus cristatus)*	European Crested Tit	8	5
*Loxia pytyopsittacus*	Parrot Crossbill	8	3
*Sylvia borin*	Garden Warbler	8	6
*Turdus iliacus*	Redwing	8	2
*Larus melanocephalus*	Mediterranean Gull	7	1
*Phylloscopus sibilatrix*	Wood Warbler	7	2
*Podiceps cristatus*	Great Crested Grebe	7	2
*Sterna dougallii*	Roseate Tern	7	2
*Sternula albifrons (Sterna albifrons)*	Little Tern	7	3
*Calidris pugnax (Philomachus pugnax)*	Ruff	6	2
*Carduelis carduelis*	Goldfinch	6	4
*Circus pygargus*	Montagu's Harrier	6	3
*Grus grus (Megalornis grus)*	Common Crane	6	2
*Regulus ignicapilla (Regulus ignicapillus)*	Firecrest	6	0
*Acrocephalus palustris*	Marsh Warbler	5	2
*Anas strepera*	Gadwall	5	2
*Bubo bubo*	Eurasian eagle owl	5	1
*Panurus biarmicus*	Bearded Tit	5	2
*Rallus aquaticus*	Water Rail	5	3
*Turdus pilaris*	Fieldfare	5	3
*Aix galericulata*	Mandarin Duck	4	2
*Anas clypeata (Spatula clypeata)*	Northern Shoveler	4	0
*Anthus petrosus*	Eurasian Rock Pipit	4	0
*Corvus cornix (Corvus corone cornix)*	Hooded Crow	4	2
*Poecile montana (Parus atricapillus)*	Willow Tit	4	1
*Porzana porzana*	Spotted Crake	4	1
*Tringa ochropus*	Green Sandpiper	4	2
*Ardea alba (Egretta alba)*	Great Egret	3	0
*Ardea purpurea*	Purple Heron	3	0
*Carduelis cabaret (Acanthis cabaret)*	Lesser Redpoll	3	1
*Chrysolophus amherstiae*	Lady Amherst's Pheasant	3	1
*Coturnix coturnix*	Common Quail	3	1
*Hippolais icterina*	Icterine Warbler	3	0
*Ixobrychus minutus*	Little Bittern	3	0
*Oriolus oriolus*	Golden Oriole	3	1
*Tringa glareola*	Wood Sandpiper	3	0
*Calidris melanotos (Erolia melanotos)*	Pectoral Sandpiper	2	1
*Columba oenas*	Stock Dove	2	1
*Locustella luscinioides*	Savi's Warbler	2	0
*Myiopsitta monachus*	Monk parakeet	2	1
*Phalaropus lobatus*	Red-necked Phalarope	2	0
*Phoenicurus ochruros*	Black Redstart	2	0
*Podiceps nigricollis*	Black-necked Grebe	2	0
*Alopochen aegyptiaca*	Egyptian Goose	1	0
*Anas acuta*	Northern Pintail	1	1
*Anas querquedula (Querquedula querquedula)*	Garganey	1	1
*Bubulcus ibis (Ardeola ibis)*	Cattle Egret	1	0
*Chrysolophus pictus*	Golden Pheasant	1	1
*Larus michahellis*	Yellow-legged Gull	1	0
*Porzana pusilla*	Baillon's Crake	1	0
*Calidris temminckii (Erolia temminckii)*	Temminck's Stint	0	0
*Eremophila alpestris (Otocorys alpestris)*	Shore (Horned) Lark	0	0
*Podiceps grisegena*	Red-necked Grebe	0	0
*Syrrhaptes paradoxus*	Pallas's Sandgrouse	0	0

Where two or more scientific names were included in the search term these are given in parenthesis.

The species with the ten highest numbers of papers were Great tit (209), Red grouse (165), Oystercatcher (156), Sparrowhawk (141), Starling (137), Blue tit (129), Guillemot (128), Lapwing (*Vanellus vanellus*, 121), Skylark (118) and Redshank (114). The top ten in terms of h-index was very similar: Great tit (52), Starling *Sturnus vulgaris* (41), Blue tit *Cyanistes caeruleus* (36), Red grouse *Lagopus lagopus* (33), Oystercatcher *Haematopus ostralegus* (33), Guillemot *Uria aalge* (33), Skylark *Alauda arvensis* (31), Sparrowhawk *Accipiter nisus* (30), Redshank *Tringa totanus* (30) and Blackbird *Turdus merula* (29). The two metrics were highly correlated (Pearson’s coefficient 0.924, p<0.001).

**Fig 2:**
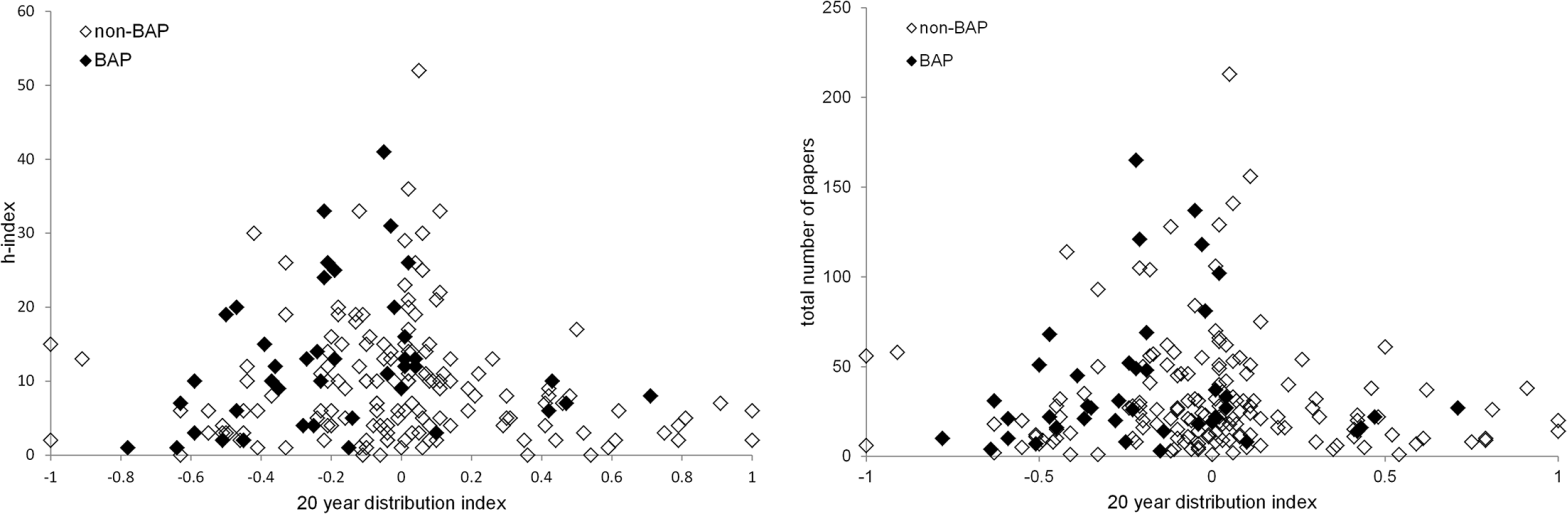
Publication metrics plotted against 20 year distribution change index (distribution change between 1988–1991 and 2008–2011 allowing for biases in recording effort; Balmer *et al*. 2013), grouped by BAP status. A positive value indicates range expansion, a negative value a contraction. Note, this index was available for 195/225 species. a) total number of papers; b) h-index.

**Fig 3:**
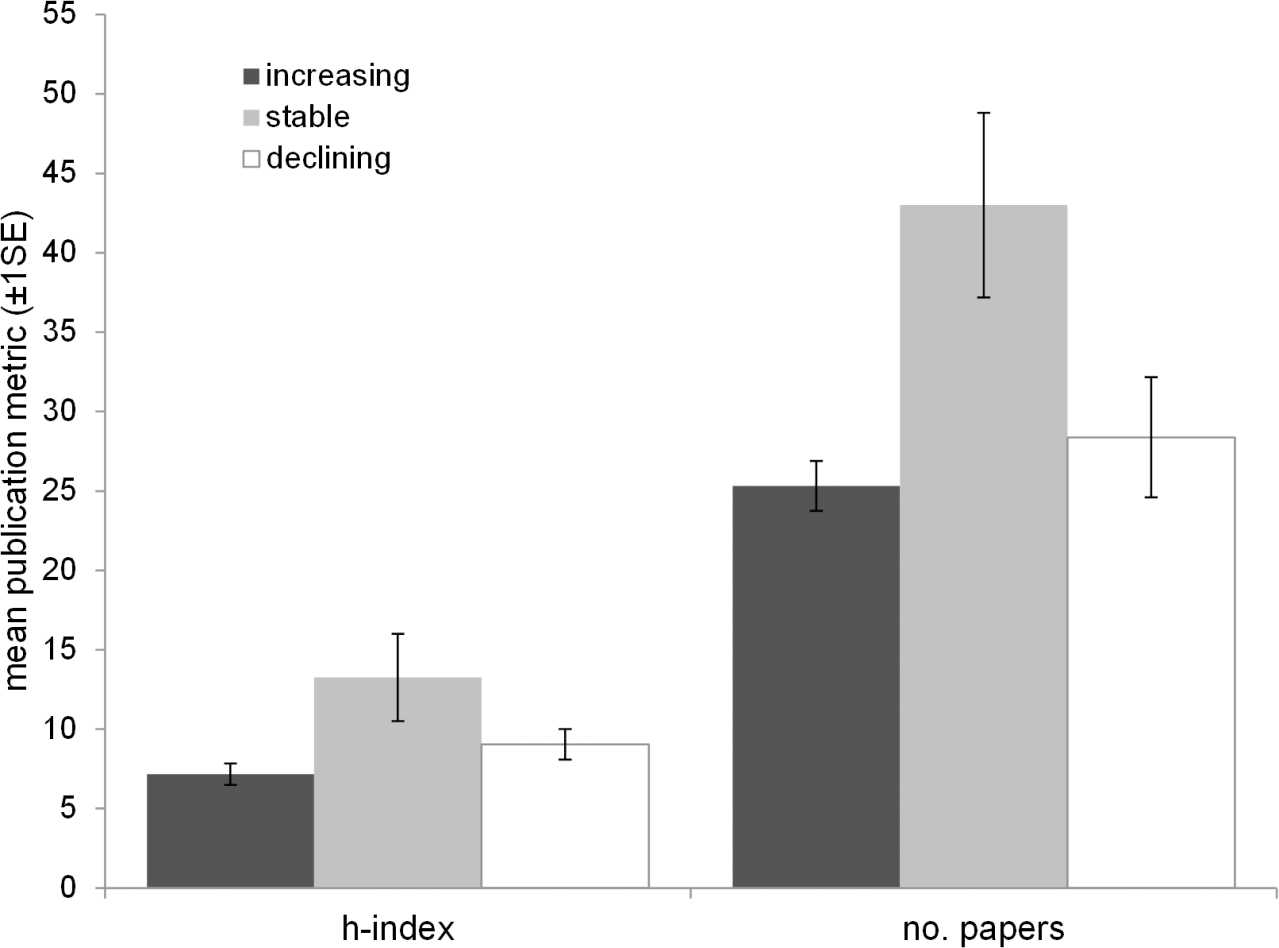
Mean publication metrics (± 1SE) in relation to changing bird distributions over the last 20 years. Species which have had stable distributions over this period have significantly higher metrics than increasing or declining species combined (no. papers: Kruskal Wallis χ^2^ = 8.71, p<0.005; h index: χ^2^ = 10.66, p<0.005).

**Fig 4:**
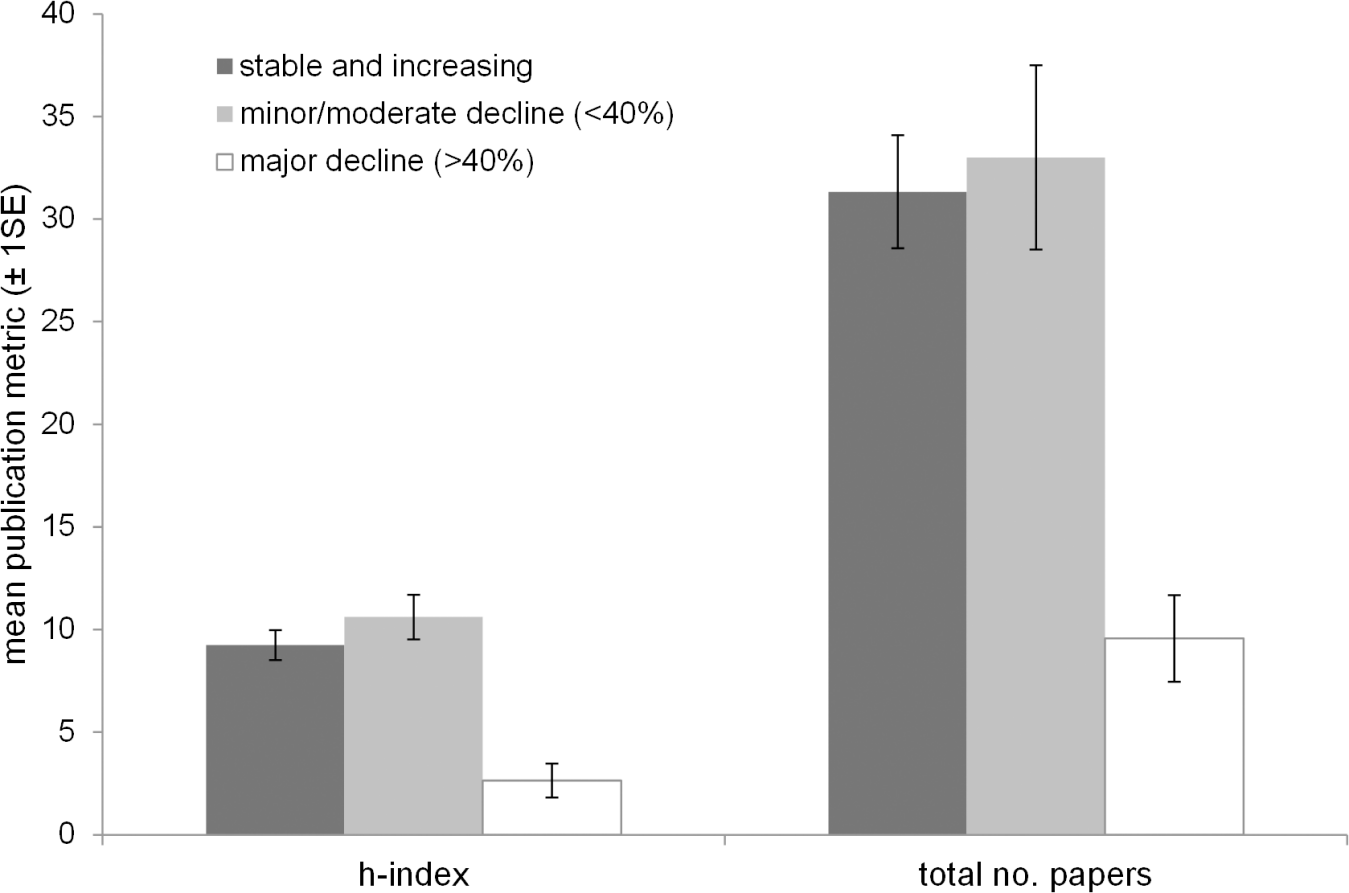
Mean publication metrics (± 1SE) in relation to changing bird distributions over the last 20 years. Species which have undergone severe declines over the past 20 years (>40%) have significantly lower publication metrics than those which have undergone either minor or moderate declines, remained stable or increased (no. papers: Kruskal Wallis χ^2^ = 14.35, p<0.001; h index: χ^2^ = 13.67, p<0.001).

The univariate comparisons showed total number of papers and h-index to be significantly lower for introduced than native species (Kruskal Wallis χ^2^ = 6.51, p<0.05 and χ^2^ = 7.58, p<0.01 respectively). BAP status was also a significant predictor of h-index, species with BAPs having typically higher h-indices (χ^2^ = 5.06, p<0.05; Fig 2B). This was not the case for total number of papers (χ^2^ = 2.57, p = 0.11; Fig 2A). Neither publication metrics were significantly influenced by Red List status (total papers: χ^2^ = 0.92, p = 0.34; h-index: χ^2^ = 0.82, p = 0.37). Breeding status (resident over migrant) had a significant effect on both metrics (total no. papers: χ^2^ = 15.33, p<0.001; h-index: χ^2^ = 12.49, p<0.001). The effect of guild was significant for h-index and close to significance for total number of papers (χ^2^ = 19.29, p<0.05; χ^2^ = 17.03, p = 0.07). There was no significant difference between the numbers of papers or h-indices for stable/increasing species and declining species (20 year trend—no. papers: χ^2^ = 0.14, p = 0.93; h-index: χ^2^ = 2.01, p = 0.37, 40 year trend–no. papers: χ^2^ = 5.17, p = 0.08; h-index: χ^2^ = 3.98, p = 0.14), however when stable populations were considered alongside increasing and declining species combined, stable populations had significantly higher metrics (stable vs. increasing and declining—no. papers: Kruskal Wallis χ^2^ = 8.71, p<0.005; h-index: χ^2^ = 10.66, p<0.005; Fig 3). Species which have undergone very severe range contractions over the last 20 years, on the other hand, (>40% contraction, [16]) have significantly lower publication metrics than those which have undergone minor to moderate declines, remained stable or increased (no. papers: χ^2^ = 14.35, p<0.001; h-index: χ^2^ = 13.67, p<0.001; Fig 4). Full outputs are given in S2 Table.

The results of the best fit multivariate models found species with higher h-indices and total numbers of papers to have significantly larger populations and distributions (total number of papers: population t value = 10.98, p<0.001; distribution t value = 9.277, p<0.001; h-index: population t value = 11.48, p<0.001; distribution t value = 9.30, p<0.001;). Guild was also a significant predictor of both number of papers and h-index (p<0.001 for both models). The ranking of groups for total number of papers was: birds of prey<gamebirds< herons/bitterns/egrets<waders<seabirds <ducks & geese<other<grebes/divers/rails< corvids<small passerines< pigeons & doves and for h-index: birds of prey<gamebirds<waders< seabirds<herons/bitterns/egrets<other<ducks & geese<small passerines<grebes/divers/ rails<corvids<pigeons & doves. Native status was also a significant predictor, native species having higher publication indices than introduced species (no. papers: t value = -3.09 p<0.005; h-index: t value = -2.59, p<0.05). Between 1972 and 2014, new papers on established introduced species appeared in the literature at 44% of the rate associated with native species (S1 Fig). BAP status was also significant, BAP species typically having higher metrics (no. papers: t value = 3.14, p<0.005; h-index: t value = 2.57, p<0.05), as was breeding status, resident breeders having higher indices than migrant breeders (no. papers: t value = -3.14, p<0.005; h-index: t value = -2.79, p<0.01). There was no significant effect of 20 year distribution trend on either metric (h-index: p = 0.689, no. papers: p = 0.203) or of Red List status (Red List: no. papers t value = 0.45, p = 0.65; h-index t value = 0.65, p = 0.52). As population and distribution were interchangeable in the model, the outputs described in this section are those from the “population” model only. Full outputs of “population” and “distribution” models can be found in S3 Table.

## Discussion

While a small body of evidence exists relating research effort to species traits [1–6], this study, alongside a similar analysis of British mammals [18], is the first attempt to systematically assess the contribution of different species to the evidence base in terms not only of research effort (total number of papers) but also “impact” (h-index). Given how important this evidence is for informing management, policy and conservation actions, this assessment is overdue.

Scientific interest in a species is a composite measure, reflecting the ease with which a species can be studied, its commercial value, the availability of research funding, species conservation status, the damage or risks associated with its presence, the personal contributions or interests of individual scientists and the public interest in the results [1,5, 18]. This range of motivations is evidenced in our ranked list of species–Great and Blue Tits for example (numbers 1 and 6 on the total number of papers list and 1 and 3 on the h-index list), rank highly as they are easy to study, populous and widely distributed, making them an excellent “model” species for the long-term testing of ecological principles. The placing of Skylark (numbers 9 and 7 respectively), in contrast, is likely a consequence of conservation status, the species having undergone steep population declines in recent decades. In addition to this, a large proportion of the work carried out on species which rank highly was undertaken by individual/teams of scientists, or university groups over long time periods—research for 60% of all papers on Great Tits, for example, was carried out at Wytham Woods, Oxford (85% of the papers which make up the h-index), while 50% of all papers on Oystercatchers include the authors Goss-Custard and/or Stillman (76% of h-index papers). While it is unsurprising that long-term studies produce larger numbers of papers than short-term studies, they also produce a greater volume of more highly cited papers, emphasising the value of long-term data sets.

While the model developed in this study cannot separate the individual drivers of research, it can identify the key factors explaining relative research effort and impact at the species level. Populous and widely distributed species, which are native rather than introduced, resident rather than migrant breeders, and are in receipt of biodiversity action plans (national-level conservation action), had statistically higher numbers of papers and h-indices and numbers of papers associated with them. Global conservation status (IUCN Red List status, [15]) was not significant.

Guild was also a significant factor, with birds of prey in receipt of most study and pigeons and doves least. Considering the wide range of research motivations discussed above, the model fit was good with only three significant outliers–Rock Pipit (*Anthus petrosus*) and Lesser Redpoll (*Carduelis cabaret*) had publication metrics lower than would be expected from their traits and Ruddy Turnstone (*Arenaria interpres*) had higher metrics. These findings are largely in agreement with those of previous studies of both birds and mammals, two global and one UK-based study [1,2,18] reporting that research effort is greater for species which have larger distribution ranges. Taxonomic status (e.g. order, family, guild) has also been shown to significantly influence research effort [2,3], as has native status, introduced mammals typically shown to be under-represented in the literature [18]. Our findings on residency status differ from those of Ducatez & Lefebvre [2], who reported that migratory bird species at the global scale had been in receipt of significantly greater research effort than resident species. Our finding may be an artefact of the relatively low numbers of migrant species in Great Britain.

H-index, used here for the first time to assess the contribution of individual bird species, is regarded by many in academic spheres as a more appropriate publication metric than alterative measures (such as total paper number) as it gives an indication of the importance or impact of papers, which other metrics do not [9]. It is also it is not influenced by the size of the ‘tail’ of less cited papers on a species [9,18] and eliminates issues associated with search engines in which the probability of identification is linked to citation number [19].

However its use does have some drawbacks. H-index necessarily invokes a “time” component–papers which have been published for longer periods of time are likely to have higher citation rates and thus h-indices. This may lead to biases in the metric results, particularly for species experiencing recent population changes or introductions. It is worth noting, however, that the number of papers published over time for different species groups within our database (raptors, farmland birds, seabirds, waders, all species,) were highly correlated with one another (0.77 to 0.93), which indicates that while biases will inevitably exist, these are broadly similar across species groups (S1 Table; S2 Fig). H-index is also more time-consuming to calculate than simple metrics such as total number of papers. In this example the two metrics were highly correlated. However, this pattern may not hold true when applied across other datasets, or for species in other regions.

Care must therefore be taken to select the correct metric to address the questions at hand. While quality (evidenced through h-index) is important in the academic arena, it is perhaps less critical in terms of policy and conservation action; papers which are used to inform policy are not necessarily those which are cited highly within academia. For this reason, total number of papers is perhaps a better indication of general interest in a species.

The outputs of the models have identified three key gaps in the bird evidence base, in terms of both paper “quality” (h-index) and “quantity” (total number of papers).

### 1) Migrant breeders

Migrant breeders in Great Britain have been in receipt of significantly less research than residents. This is a significant omission considering many migrant breeders have undergone severe population declines in recent decades (e.g. Turtle Dove *Streptopelia turtur*, [20]; European Nightjar *Caprimulgus europaeus*, [21]). This is highlighted by recently published distribution change data [16], which shows that a higher percentage of migrant breeders have undergone range contractions over both the last 20 and 40 years than resident breeders (20 years: 35% of residents have declined vs. 46% of migrants; 40 years: residents 39% vs. migrants 52%). The reasons why migrant breeders are understudied are likely to be multifold, and include difficulty of study (by their nature, migrants are unavailable for study for long periods of the year), fewer species to study (72 migrant breeders vs. 153 residents), as well as small distributions/populations of some key migrant species (mean distribution of migrants vs. residents is 23.78 (±3.51) vs. 39.96 (±2.68) % of occupied 10km squares, and for population size 245,104 (±81,774) vs. 909,461 (± 195,084) individuals [13,16]).

### 2) Introduced species

Introduced species had disproportionally lower h-indices and numbers of papers associated with them than native species, even when allowing for differences in distribution and population. This is a concern when the negative effect of many introduced species on ecosystems is considered [22] and that introduced species often conflict with both native species and human interests (e.g. Canada Goose *Branta canadensis* [23], Ring-necked Parakeet *Psittacula krameri* [24], Ruddy Duck *Oxyura jamaicensis* [25]). It is also a significant result given that a number of introduced species are commercially important (e.g. Red-legged Partridge *Alectoris rufa* and Pheasant *Phasianus colchicus*) and have been present for a long time. For some introduced species, their low ranking in the list may be a result of their relatively recent introduction into the country–sufficient time simply has not elapsed for a large body of work to have amassed. Alternatively, some species may be viewed as existing at sufficiently low populations to pose much risk on the larger scale (e.g. Eagle Owl). Other species, such as Little Owl (*Athene noctua*), are long-term breeders yet do not appear problematic; therefore there is little interest in their study. However, with increases in the distribution of several species known to cause conflicts in other countries (e.g. Egyptian Goose *Alopochen aegyptiaca* in South Africa [26]: 163% increase in last 20 years, Monk parakeet in Spain [27]: 50% increase in last 20 years [16]), these knowledge gaps may limit effective policy or management.

### 3) Conservation status—BAP status vs. distribution trends

Species with biodiversity action plans tended to have higher numbers of papers and h-indices than those without plans. This is encouraging as it indicates that conservation status and research effort are linked (although whether conservation status is driving research or vice versa cannot be determined by this analysis). However, BAP is only one aspect of conservation status (national action)–Red List status (international action) did not have any significant impact on either publication metric. Moreover, species which have undergone distribution contractions over the last 20 or 40 years do not have significantly higher publication metrics. Indeed, looking at overall patterns, it is species with stable distributions which have been in receipt of most research over the last 20 years (Fig 3). Moreover, species which have undergone very severe range contractions over the last 20 years (>40% [16] have significantly lower publication metrics than those which have undergone minor to moderate declines, remained stable or increased ((Fig 4; although this is clearly not the case for all declining species, for example Skylark).

These findings may reflect the difficulty of studying scarce or declining species, however they do raise questions about the relative strength of the evidence base that underpins conservation actions for such species. However as this study does not take account of time, and research motivations have undoubtedly changed in recent decades, this finding may not accurately reflect current British research priorities.

Therefore, while it is encouraging that BAP species appear to be in receipt of more and better quality study than non-BAP species, care should be taken to ensure that other declining species which do not benefit from BAP also receive sufficient research effort. It is likely that funding is harder to obtain for species not badged in this way.

## Conclusions and Wider Applications

In summary, this paper has produced ranked lists of species based on their publication metrics. While their position on these lists results from a variety of factors, the relatively simple model we have constructed provides a very good fit to the data. While this method has been applied to birds in this instance, it could and, we believe, should be repeated for other taxa or species groups.

The results of this work have raised a number of questions which warrant further study—1) How have research motivations changed over time? Is there evidence of a change away from theory-led research towards conservation or policy led-research in recent years? 2) How have changes in conservation status and/or policy impacted upon publication rates?; and 3) Are these changes cause or effect? Is policy being led by research or research by policy?

In short, we believe this to be an exciting and useful approach to understanding human- introduced biases in the quality and quantity of scientific literature at a species level, something which will help provide a solid foundation for both conservation and evidence-based policy-making in the future.

## Supporting Information

S1 Database Database of all papers included in the analysis. (XLSX)

S1 Fig The mean cumulative number of papers published per native and introduced species over the period 1973–2014. The solid line shows native species and the dashed line introduced species.
(TIF)

S2 Fig Series of graphs showing the relationship of numbers of papers published against time (1972–2014. A) All species; B) Raptors; C) Farmland Species; D) Seabirds; E) Waders.
(ZIP)

S1 Table List of model co-variates and their sources. (DOCX)

S2 Table Results of Kruskal-Wallis tests comparing total number of papers and h-index with five key factors. (DOCX)

S3 Table Output from best fit models for both publication metrics (total number of papers and h-index). A) Total number of papers and population; B) Total number of papers and distribution; C) h-index and population; D) h-index and distribution.
(DOCX)

S4 Table Pearson correlation coefficients for numbers of papers across time for a range of species groups/all species. (DOCX)

## References

[1] Brooke ZM, Bielby J, Nambiar K, Carbone C. Correlates of Research Effort in Carnivores: Body Size, Range Size and Diet Matter. PLoS ONE 2014; 9(4): e93195 doi: 10.1371/journal.pone.0093195 24695422 10.1371/journal.pone.0093195 PMC3973602

[2] Ducatez S, Lefebvre L. Patterns of research effort in birds. PLoS One. 2014; 9(2): e89955 doi: 10.1371/journal.pone.0089955 24587149 10.1371/journal.pone.0089955 PMC3935962

[3] Brito D, Oprea M. Mismatch of research effort and threat in avian conservation biology. Trop Conserv Sci. 2009; 2(3): 353–362.

[4] Brodie JF. Is research effort allocated efficiently for conservation? Felidae as a global case study. Biodivers Conserv. 2009; 19:2927–2939.

[5] Bonnet X, Shine R, Lourdais O. Taxonomic chauvinism. Trends Ecol Evol 2002; 17(1): 1–3.

[6] Clark AJ, May RM. Taxonomic bias in conservation research. Science 2002; 297:191–192. 12117005 10.1126/science.297.5579.191b

[7] RSPB. RSPB website. Available: http://www.rspb.org.uk/about/facts.aspx. Accessed 8^th^ July 2014.

[8] Wilkin TA, Garant D, Gosler AG, Sheldon BC. Density effects on life-history traits in a wild population of the great tit Parus major: analyses of long-term data with GIS techniques. J Appl Ecol; 2006 75: 604–615. 10.1111/j.1365-2656.2006.01078.x 16638013

[9] Hirsch JE. An index to quantify an individual's scientific research output. PNAS 2005; 102: 16569–16572. 16275915 10.1073/pnas.0507655102 PMC1283832

[10] Malesios C, Psarakis S. Comparison of the h-index for different fields of research using bootstrap methodology. Qual Quan. 2014; 48: 521–545.

[11] Harrop AHJ, Collinson JM, Martin J, Dudley SP, Kehoe C. The British List: A Checklist of Birds of Britain (8th edition). Ibis 2013; 155: 635–676.

[12] Fera. Overview of conflicts between human and wildlife interests in the UK. Report to Defra; 2010.

[13] BTO. BTO BirdFacts website. Available: http://www.bto.org/about-birds/birdfacts. Accessed 2014 Jul 8.

[14] JNCC. UK BAP priority species and habitats. Available: http://jncc.defra.gov.uk/page-5705. Accessed 2014 Jul 8.

[15] IUCN. The IUCN Red List of Threatened Species. Available: http://www.iucnredlist.org/. Accessed 2014 Jul 8.

[16] Balmer DE, Gillings S, Caffrey BJ, Swann RL, Downie IS, Fuller RJ. Bird Atlas 2007–11: the breeding and wintering birds of Britain and Ireland BTO Books, Thetford, UK 720 p; 2013.

[17] EOL. Traitbank–Encyclopaedia of Life. Available: http://eol.org/info/516. Accessed 2014 Jun 18.

[18] Robertson PA, McKenzie AJ. The scientific profiles of British terrestrial mammals as measured by citation rates. Mammal Review; 2015 9(4), e93195.

[19] Bar-Ilan J. Which h-index? A comparison of WoS, Scopus and Google Scholar. Scientometrics; 2008 74: 257–271.

[20] Dunn JC, Morris AJ. Which features of UK farmland are important in retaining territories of the rapidly declining Turtle Dove *Streptopelia turtur*? Bird Study 2012; 59: 394–402.

[21] Langston RHW, Wotton SR, Conway GJ, Wright LJ, Mallord JW, Currie FA, et al Nightjar *Caprimulgus europaeus* and Woodlark *Lullula arborea*—Recovering species in Britain? Ibis 2007; 149: 250–260.

[22] Manchester SJ, Bullock JM. The impacts of non-native species on UK biodiversity and the effectiveness of control. J Appl Ecol. 2001; 37: 845–864.

[23] Baxter AT, Robinson AP. Monitoring and influencing feral Canada goose (*Branta canadensis*) behaviour to reduce birdstrike risks to aircraft. Int J Pest Manage 2007; 53: 341–346.

[24] Peck HL, Pringle HE, Marshall HH, Owens IPF, Lord AM. Experimental evidence of impacts of an invasive parakeet on foraging behaviour of native birds. Behav Ecol. 2014; 25: 582–590. 24822022 10.1093/beheco/aru025 PMC4014307

[25] Smith GC, Henderson IS, Robertson PA. A model of ruddy duck *Oxyura jamaicensis* eradication for the UK. J Appl Ecol. 2005; 42: 546–555.

[26] Mackay B, Little RM, Amar A, Hockey PAR. Incorporating environmental considerations in managing Egyptian Geese on golf courses in South Africa. J Wildlife Manage. 2014; 78: 671–678.

[27] Rodriguez-Pastor R, Senar JC, Ortega A, Faus J, Uribe F, Montalvo T. Distribution patterns of invasive Monk parakeets (*Myiopsitta monachus*) in an urban habitat. Anim Biodiver Conserv. 2012; 35: 107–117.

