# Supplementary figures and images for: Which Species Are We Researching and Why? A Case Study of the Ecology of British Breeding Birds
Source: PLoS One. 2015 Jul 8;10(7):e0131004. doi: 10.1371/journal.pone.0131004 (PMC4496060; doi:10.1371/journal.pone.0131004)

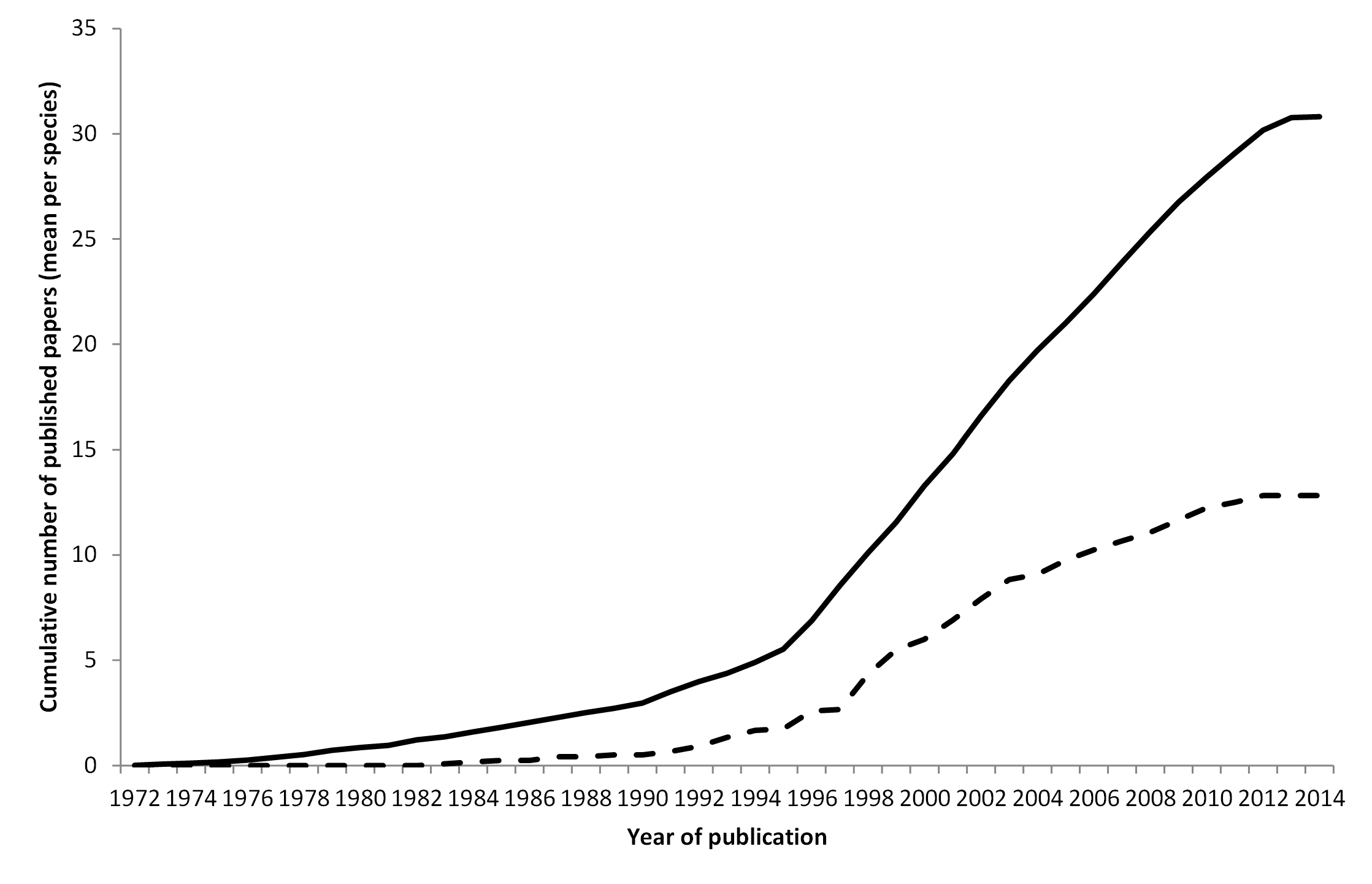

Supplement: S1 Fig — The solid line shows native species and the dashed line introduced species. (TIF) [file pone.0131004.s002.tif]

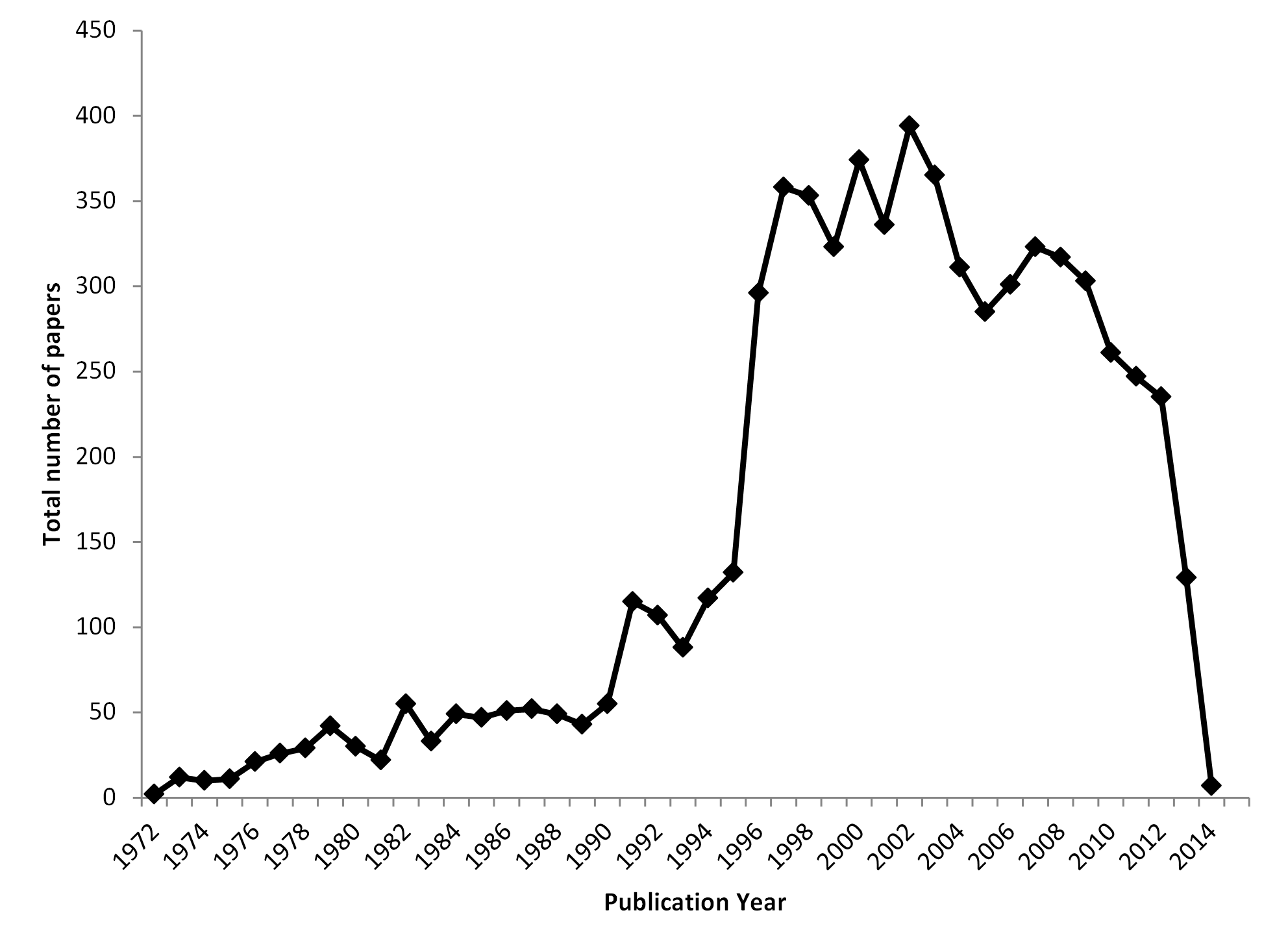

Supplement: S2 Fig — A) All species; B) Raptors; C) Farmland Species; D) Seabirds; E) Waders. (ZIP) [file pone.0131004.s003.zip › S2A_Fig.tif]

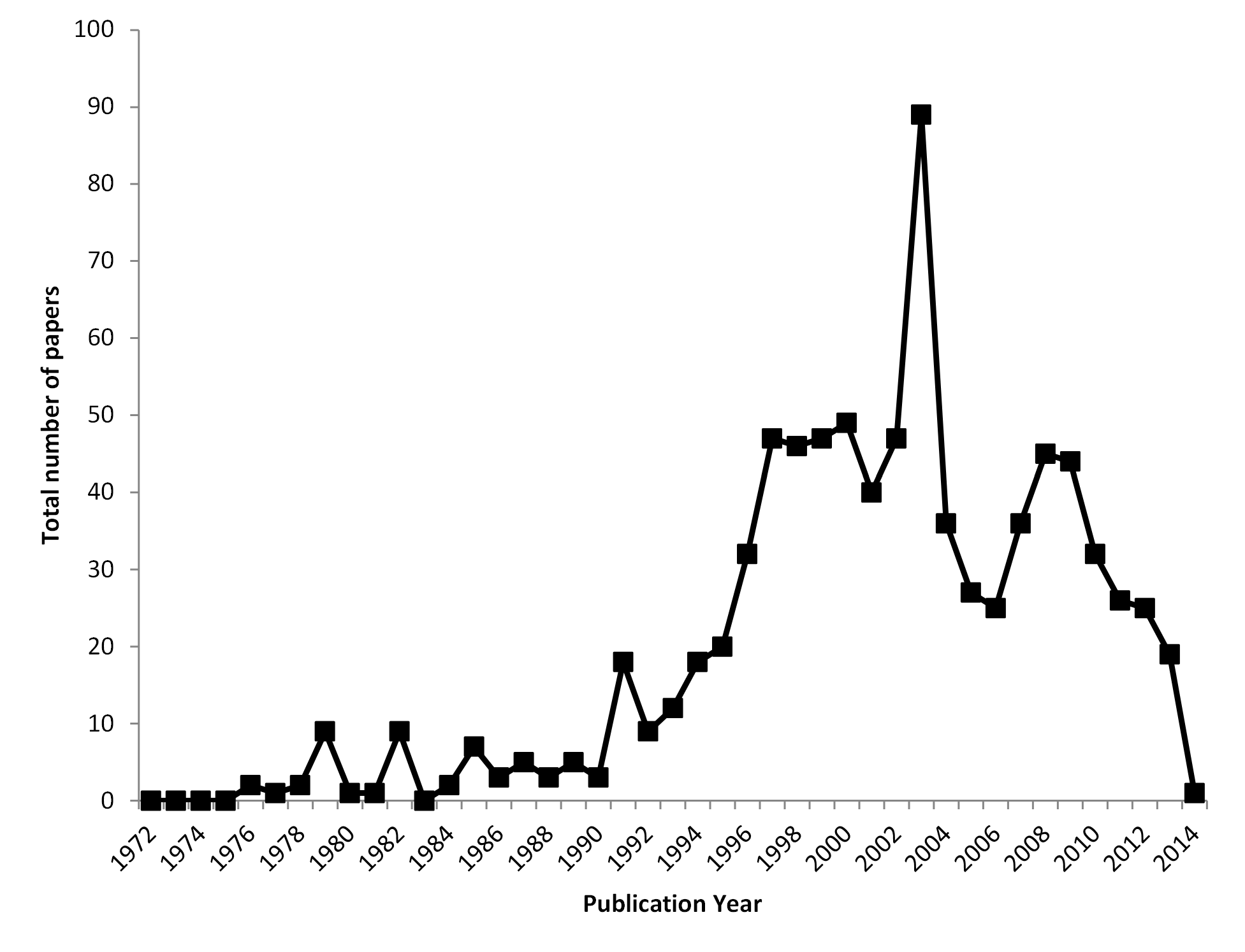

Supplement: S2 Fig — A) All species; B) Raptors; C) Farmland Species; D) Seabirds; E) Waders. (ZIP) [file pone.0131004.s003.zip › S2B_Fig.tif]

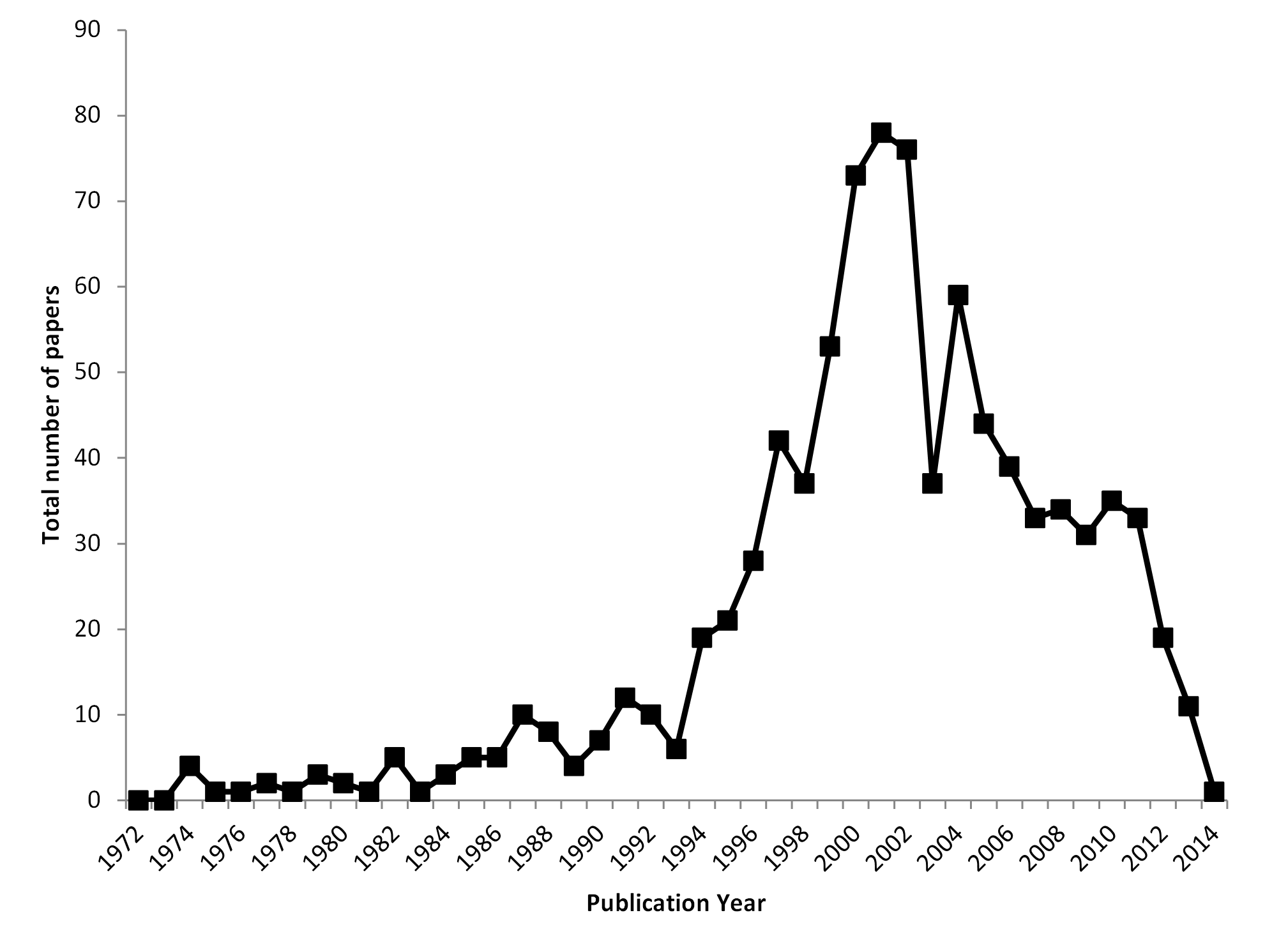

Supplement: S2 Fig — A) All species; B) Raptors; C) Farmland Species; D) Seabirds; E) Waders. (ZIP) [file pone.0131004.s003.zip › S2C_Fig.tif]

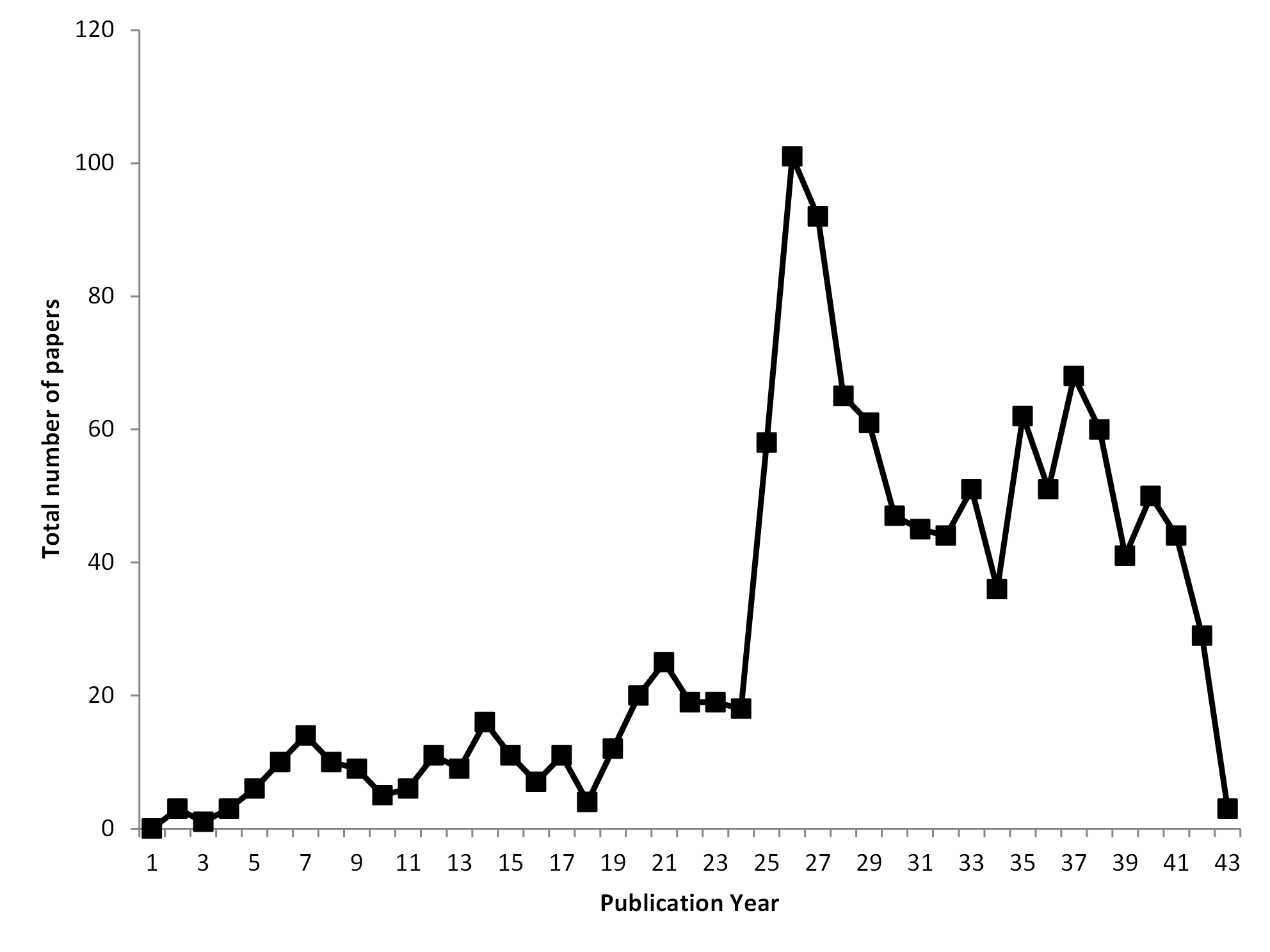

Supplement: S2 Fig — A) All species; B) Raptors; C) Farmland Species; D) Seabirds; E) Waders. (ZIP) [file pone.0131004.s003.zip › S2D_Fig.tif]

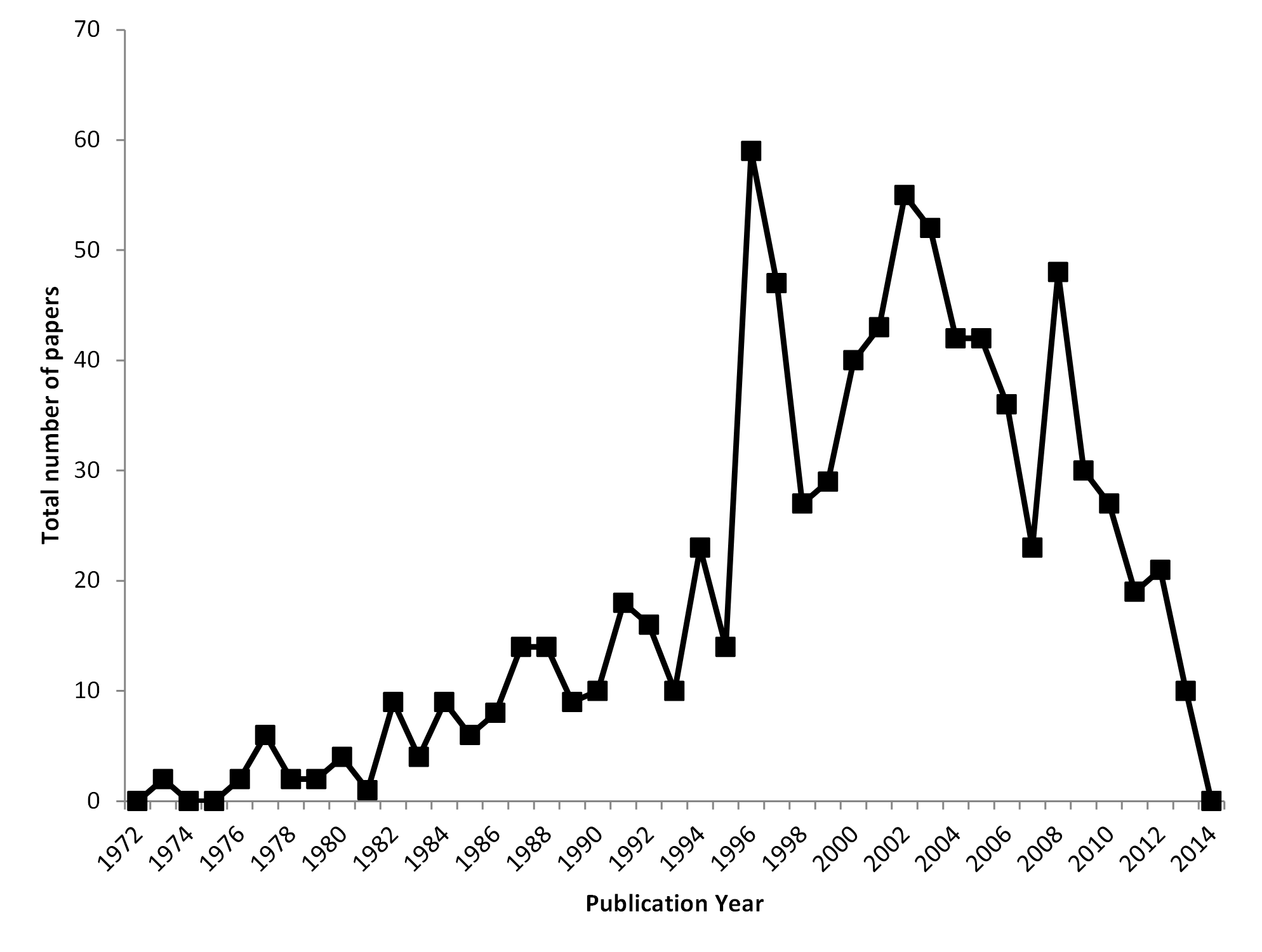

Supplement: S2 Fig — A) All species; B) Raptors; C) Farmland Species; D) Seabirds; E) Waders. (ZIP) [file pone.0131004.s003.zip › S2E_Fig.tif]
