# Supplementary material for: Which Species Are We Researching and Why? A Case Study of the Ecology of British Breeding Birds
Source: PLoS One. 2015 Jul 8;10(7):e0131004. doi: 10.1371/journal.pone.0131004 (PMC4496060; doi:10.1371/journal.pone.0131004)
Supplement: S1 Table — (DOCX) [file pone.0131004.s004.docx]

Table S1. List of co-variates and their sources. Note, not all co-variates were used in the final models.

| Predictor | Description/levels | | Source |
| --- | --- | --- | --- |
| ***Continuous variables*** | | |  |
| 1) Distribution | | % 10km squares occupied in England, Scotland and Wales (1960-2013) | 224/225 species: Data obtained from Bird Atlas 2007-11 (Balmer *et al.*. 2013). Total distribution (possible, probable and confirmed breeding areas combined). Where distribution was recorded as “<1%” a value of 0.5 was used. Monk parakeet: data obtained from AHVLA (pers. comm. P. Robertson). |
| 2) Population | | Estimated number of individuals | 223/225 species: BTO Birdfacts (http://www.bto.org/about-birds/birdfacts). Eagle owl: estimate from Elkins, 2010; Monk parakeet: data obtained from AHVLA (pers. comm. P. Robertson). |
| 3) Body weight | | Mean body weight (kg) | Majority of species: BTO Birdfacts (http://www.bto.org/about-birds/birdfacts). Additional species: TraitBank (EOL, 2014). |
| 4) 20 year index, 20 year change and 40 year change | | 20 year index: distribution change (number of 10km squares occupied) between 1988-1991 and 2008-2011 atlases, allowing for biases in recording effort.  20 year change: percentage change in distribution between 1988-1991 and 2008-2011.  40 year change: percentage change in distribution between 1968-1972 and 2008-2011.  Positive and negative values indicate range expansion and contraction respectively (Balmer *et al.*. 2013 p. 154) | 20-year index: available for 195 spp  20-and 40 year change: available for 221 spp. All Balmer *et al.*, 2013 |
| ***Categorical factors*** | | |  |
| Introduced? | Yes/no | | 224 spp: BTO BirdFacts and/or Balmer *et al.* 2013. Monk parakeet: Non-Native Species Secretariat factsheet (NNSS, 2014). |
| Functional group | Ducks & geese (22 spp), herons/bitterns/egrets (10 spp), game birds (10 spp), corvids & small passerines (6 & 80 spp), birds of prey (21 spp), seabirds (23 spp), doves & pigeons (6 spp), grebes/divers/rails (14 spp), waders (24 spp) and other (9 spp). | | All species: IUCN Red List (IUCN, 2014), with further groupings carried out subjectively. |
| Breeding status | Resident or migrant | | 223 spp: Harrop *et al.* 2013. Eagle owl: Balmer *et al*. 2013, Monk parakeet: NNSS, 2014. |
| Conservation status | | |  |
| UK | BAP Yes/no | | JNCC, 2014 |
| Elsewhere | Red List: Not Assessed, Least Concern, Near Threatened. | | IUCN, 2014 |
