# Supplementary material for: Which Species Are We Researching and Why? A Case Study of the Ecology of British Breeding Birds
Source: PLoS One. 2015 Jul 8;10(7):e0131004. doi: 10.1371/journal.pone.0131004 (PMC4496060; doi:10.1371/journal.pone.0131004)
Supplement: S2 Table — (DOCX) [file pone.0131004.s005.docx]

Table S2. Results of Kruskal-Wallis tests comparing total number of papers and h index with five key factors. Native, BAP, Red List and breeding status were all 2 level factors (native vs. introduced, BAP vs. non-BAP, Red List status “Not assessed” or “Least concern” vs. “Near threatened”). 20 and 40 year distribution trend were three level factors (increase, stable, decline). Functional group was an 11 level factor - ducks and geese (22 species), herons/bitterns /egrets (10 species), gamebirds (10 species), corvids and small passerines (6 and 80 spp), birds of prey (21 species), seabirds (23 species), doves and pigeons (6 species), grebes/divers/rails (14 species) and waders (24 species) and other (9 species). Introduced vs. native status was significant for both metrics, BAP status for h index but not for total number of papers. Neither distribution status nor Red List status was not significant for either of the metrics while breeding status and functional group were significant or close to significance for both metrics.

|  | Total no. papers | h-index |
| --- | --- | --- |
| Native status | χ^2^=6.51, p<0.05 * | χ^2^=7.58, p< 0.01 ** |
| BAP status | χ^2^=2.57, p=0.11 | χ^2^=5.06, p<0.05 * |
| Red List | χ^2^=0.92, p=0.34 | χ^2^=0.82, p=0.37 |
| Breeding status | χ^2^= 15.33, p<0.001 *** | χ^2^=12.49, p<0.001 *** |
| 20yr distribution | χ^2^=0.14, p=0.93 | χ^2^= 2.01, p=0.37 |
| 40yr distribution | χ^2^= 5.17, p=0.08 | χ^2^= 3.98 p=0.14 |
| Functional group | χ^2^=17.03, p=0.07 | χ^2^=19.29, p<0.05 * |
