# Supplementary material for: Which Species Are We Researching and Why? A Case Study of the Ecology of British Breeding Birds
Source: PLoS One. 2015 Jul 8;10(7):e0131004. doi: 10.1371/journal.pone.0131004 (PMC4496060; doi:10.1371/journal.pone.0131004)
Supplement: S3 Table — A) Total number of papers and population; B) Total number of papers and distribution; C) h-index and population; D) h-index and distribution. (DOCX) [file pone.0131004.s006.docx]

Table S3. Output from best fit models for both publication metrics. Metrics for each species were entered as the response variable into general linear models in R (version 3.0.0). Population size and distribution (% 10km squares) were correlated and interchangeable in the model (Pearson’s coefficient 0.508, p<0.001) and included as a continuous variable (log10+1 transformed). Five factors were also included in the model describing species functional group (birds of prey, ducks and geese, gamebirds, grebes/divers/rails, herons/bitterns/egrets, corvids, “other”, pigeons and doves, seabirds, small passerines and waders, whether the species was introduced or native, the subject of a Biodiversity Action Plan (BAP), its Red List status (“not assessed” and “least concern” vs. “near threatened”) and breeding status (resident or migrant). Models were run with population and distribution separately for each metric. A) Total number of papers and population; B) Total number of papers and distribution; C) h-index and population; D) h-index and distribution

A)

|  | Estimate | Std. Error | t value | Pr(>\|t\|) |
| --- | --- | --- | --- | --- |
| (Intercept) | 1.31706 | 0.26046 | 5.057 | 9.51x10^-7^ *** |
| population | 0.17256 | 0.01571 | 10.981 | 2.00x10^-16^ *** |
| ducks and geese | -0.35915 | 0.10218 | -3.515 | 0.000543 *** |
| gamebirds | -0.15098 | 0.13661 | -1.105 | 0.270379 |
| grebes/divers/rails | -0.45001 | 0.11815 | -3.809 | 0.000185 *** |
| herons/bitterns/egrets | -0.1542 | 0.12951 | -1.191 | 0.235169 |
| corvids | -0.46517 | 0.15786 | -2.947 | 0.003588 ** |
| other | -0.36193 | 0.13622 | -2.657 | 0.008511 ** |
| pigeons and doves | -0.77238 | 0.17096 | -4.518 | 1.06x10^-5^ *** |
| seabirds | -0.22045 | 0.10463 | -2.107 | 0.036353 * |
| small passerines | -0.4672 | 0.08835 | -5.288 | 3.18x10^-7^ *** |
| waders | -0.17759 | 0.1029 | -1.726 | 0.085891 |
| red list | 0.07872 | 0.17303 | 0.455 | 0.649643 |
| BAP | 0.1988 | 0.06325 | 3.143 | 0.001923 ** |
| introduced | -0.34982 | 0.11305 | -3.094 | 0.00225 ** |
| breeding status | -0.16543 | 0.05266 | -3.142 | 0.001931 ** |
| 20yr distribution (stable) | -0.02579 | 0.08214 | -0.314 | 0.753889 |
| 20yr distribution (decline) | 0.07904 | 0.05354 | 1.476 | 0.141427 |

B)

|  | Estimate | Std. Error | t value | Pr(>\|t\|) |
| --- | --- | --- | --- | --- |
| (Intercept) | 1.379901 | 0.277225 | 4.978 | 1.37E-06 *** |
| distribution | 0.411997 | 0.044412 | 9.277 | 2.00E-16 *** |
| ducks and geese | -0.26462 | 0.107939 | -2.452 | 0.01507 * |
| gamebirds | -0.04861 | 0.144219 | -0.337 | 0.7364 |
| grebes/divers/rails | -0.39121 | 0.125544 | -3.116 | 0.0021 ** |
| herons/bitterns/egrets | 0.001303 | 0.139189 | 0.009 | 0.99254 |
| corvids | -0.28264 | 0.164608 | -1.717 | 0.08749 |
| other | -0.25729 | 0.143235 | -1.796 | 0.07394 |
| pigeons and doves | -0.55564 | 0.177612 | -3.128 | 0.00202 ** |
| seabirds | 0.15898 | 0.10914 | 1.457 | 0.14676 |
| small passerines | -0.27712 | 0.090013 | -3.079 | 0.00237 ** |
| waders | -0.04519 | 0.109359 | -0.413 | 0.6799 |
| red list | 0.12748 | 0.18322 | 0.696 | 0.48737 |
| BAP | 0.170044 | 0.066765 | 2.547 | 0.01161 * |
| introduced | -0.33704 | 0.120062 | -2.807 | 0.00548 ** |
| breeding status | -0.18454 | 0.055647 | -3.316 | 0.00108 ** |
| 20yr distribution (stable) | -0.07738 | 0.087285 | -0.887 | 0.37638 |
| 20yr distribution (decline) | 0.001917 | 0.056524 | 0.034 | 0.97297 |

C)

|  | Estimate | Std. Error | t value | Pr(>\|t\|) |
| --- | --- | --- | --- | --- |
| (Intercept) | 0.68 | 0.231 | 2.944 | 0.003621 ** |
| population | 0.15993 | 0.01394 | 11.476 | 2.00x10^-16^ *** |
| ducks and geese | -0.2572 | 0.09062 | -2.838 | 0.004999 ** |
| gamebirds | -0.01476 | 0.12116 | -0.122 | 0.903144 |
| grebes/divers/rails | -0.29989 | 0.10478 | -2.862 | 0.004651 ** |
| herons/bitterns/egrets | -0.1773 | 0.11486 | -1.544 | 0.12422 |
| corvids | -0.32706 | 0.14001 | -2.336 | 0.020463 |
| other | -0.23633 | 0.12081 | -1.956 | 0.051811 * |
| pigeons and doves | -0.54196 | 0.15162 | -3.575 | 0.000438 *** |
| seabirds | -0.13447 | 0.09279 | -1.449 | 0.148839 |
| small passerines | -0.26836 | 0.07835 | -3.425 | 0.000744 *** |
| waders | -0.05694 | 0.09126 | -0.624 | 0.533347 |
| red list | 0.09997 | 0.15346 | 0.651 | 0.515502 |
| BAP | 0.14436 | 0.0561 | 2.573 | 0.010785 * |
| introduced | -0.25937 | 0.10026 | -2.587 | 0.010381 * |
| breeding status | -0.13048 | 0.0467 | -2.794 | 0.005704 ** |
| 20yr distribution (stable) | -0.04261 | 0.07285 | -0.585 | 0.559267 |
| 20yr distribution (decline) | 0.01619 | 0.04748 | 0.341 | 0.733536 |

D)

|  | Estimate | Std. Error | t value | Pr(>\|t\|) |
| --- | --- | --- | --- | --- |
| (Intercept) | 0.76017 | 0.24989 | 3.042 | 0.00266 ** |
| distribution | 0.37216 | 0.04003 | 9.296 | 2.00x10^-16^ *** |
| ducks and geese | -0.17017 | 0.0973 | -1.749 | 0.0818 |
| gamebirds | 0.08006 | 0.13 | 0.616 | 0.53869 |
| grebes/divers/rails | -0.24824 | 0.11316 | -2.194 | 0.0294 * |
| herons/bitterns/egrets | -0.03879 | 0.12546 | -0.309 | 0.75749 |
| corvids | -0.15404 | 0.14838 | -1.038 | 0.30042 |
| other | -0.13726 | 0.12911 | -1.063 | 0.28899 |
| pigeons and doves | -0.3362 | 0.1601 | -2.1 | 0.03697 * |
| seabirds | 0.21401 | 0.09838 | 2.175 | 0.03076 * |
| small passerines | -0.09007 | 0.08114 | -1.11 | 0.26829 |
| waders | 0.06341 | 0.09858 | 0.643 | 0.52077 |
| red list | 0.14352 | 0.16515 | 0.869 | 0.38587 |
| BAP | 0.11702 | 0.06018 | 1.944 | 0.05323 . |
| introduced | -0.25156 | 0.10822 | -2.324 | 0.02109 * |
| breeding status | -0.15147 | 0.05016 | -3.02 | 0.00285 ** |
| 20yr distribution (stable) | -0.08858 | 0.07868 | -1.126 | 0.26155 |
| 20yr distribution (decline) | -0.05464 | 0.05095 | -1.072 | 0.28483 |
