# Supplementary material for: Which Species Are We Researching and Why? A Case Study of the Ecology of British Breeding Birds
Source: PLoS One. 2015 Jul 8;10(7):e0131004. doi: 10.1371/journal.pone.0131004 (PMC4496060; doi:10.1371/journal.pone.0131004)
Supplement: S4 Table — (DOCX) [file pone.0131004.s007.docx]

S4 Table Pearson’s correlation coefficient relating a range of species groups with the number of papers published by year (1972-2014).

|  | All species | Raptors | Farmland species (FBI) | Seabirds |
| --- | --- | --- | --- | --- |
| Raptors | 0.930; p<0.001 | x | x | x |
| Farmland species (FBI*) | 0.914; p<0.001 | 0.816; p<0.001 | x | x |
| Seabirds | 0.918; p<0.001 | 0.825; p<0.001 | 0.766; p<0.001 | x |
| Waders | 0918; p<0.001 | 0.864; p<0.001 | 0.856; p<0.001 | 0.805; p<0.001 |

* FBI: Farmland Bird Index species includes 19 species of birds that are dependent on farmland, and not able to thrive in other habitats (Defra, 2014; available at <https://www.gov.uk/government>/ statistics/wild-bird-populations-in-the-uk (accessed on 28/01/15))
